# Characterization of oncogenes on chromosome 21 identified by shRNA-based viability screening

**DOI:** 10.1186/2194-7791-1-S1-A20

**Published:** 2014-09-11

**Authors:** Lena Stachorski, Veera Raghavan Thangapandi, Dirk Reinhardt, Jan-Henning Klusmann

**Affiliations:** Department of Pediatric Hematology and Oncology, Hannover Medical School, Hannover, Germany

Children with trisomy 21 (Down syndrome, DS) are predisposed to develop acute megakaryoblastic leukemia (DS-AMKL) as well as the antecedent transient leukemia (DS-TL). Mutations in the transcription factor GATA1 have been found in nearly all children with DS-AMKL and DS-TL, but not in other malignancies. Recent whole genome sequencing efforts suggested that the interplay of trisomy 21 and GATA1s-mutation is sufficient to cause DS-TL.

To decipher the deregulated oncogenic gene network on hsa21, we conducted a lentiviral shRNA-based viability screening using *GATA1s*-mutated DS-AMKL cell line CMK as well as non-DS-AML cell lines (K562, M-07). Knock-down of 42 genes conferred a profound selective growth disadvantage in DS-AMKL cell lines. 31 candidate genes are located in chromosomal region 21q22.1-21q22.3. 11 (out of 14 tested) were overexpressed in DS-AMKL compared to non-DS-AMKL. A secondary functional validation screening revealed that the potential oncogenes participate in different cellular processes affecting proliferation, cell viability, apoptosis or differentiation.

To further delineate the impact of 11 selected candidates on normal hematopoiesis, we characterized their effects in gain- and loss-of-function studies using CD34^+^ hematopoietic stem and progenitor cells (HSPCs). Knockdown of four genes (*USP25*, *BACH1*, *U2AF1* and *C21orf33*) inhibited megakaryocytic and erythroid in vitro differentiation, while enhancing myeloid differentiation. The myeloid differentiation bias was also observed for *ATP5O* and *C21orf45.* The opposite effect was observed in gain-of-function studies. Ectopic expression of six genes (*U2AF1*, *C21orf33*, *IFNGR2*, *WDR4* or *GABPA*) resulted in a switch from erythroid to megakaryocytic differentiation.

Thus, we propose a complex interactive network located on hsa21. Deregulation of this network might result in synergistic effects on hematopoietic differentiation, which promotes transformation of GATA1s-mutated fetal hematopoietic progenitor cells.

